# A Potential Role of Intraoperative Lidocaine in Patients Undergoing Thyroidectomy—An Original Systematic Review and Meta‐Analysis

**DOI:** 10.1002/hsr2.71675

**Published:** 2025-12-22

**Authors:** Ayesha Khan, Syeda Emmama, Mahnoor Aamir, Munazzah Marium, Iftikhar Rashid, Marium Abbas, Aleezah Fatima, Alina Amir, Shafin Bin Amin, Syed Ali Arsal, Umer Iqbal, Muhammad Maaz, Aashish Kumar, Inibehe Ime Okon

**Affiliations:** ^1^ Dow Medical College Mission Rd Karachi City Sindh Pakistan; ^2^ Islamabad Medical and Dental College Q645 + 2XP Wadi‐ul‐Ilm Islamabad Islamabad Capital Territory Pakistan; ^3^ Jinnah Sindh Medical University Karachi Pakistan; ^4^ Shaheed Mohtarma Benazir Bhutto Medical College Karachi Pakistan; ^5^ Department of Research Medical Research Circle (MedReC) Bukavu Democratic Republic of the Congo

**Keywords:** analgesic efficacy, lidocaine, meta‐analysis, postoperative pain, thyroidectomy

## Abstract

**Introduction:**

Thyroidectomy is a routine operation for diverse thyroid diseases between benign and malignant. In such cases, the complications and postoperative pains, such as nausea, vomiting, and hemodynamic instability, complicate recovery and lengthen hospital stays. Lidocaine may prove beneficial due to its analgesic, anti‐inflammatory, and antihyperalgesic actions. The current systematic review and meta‐analysis assessed the effect of IV lidocaine infusion on postoperative pain and its complications in patients undergoing total thyroidectomy.

**Methods:**

A systematic review of published RCTs between 2016 and 2023 was conducted using PubMed, Embase, and Google Scholar. Their inclusion criteria were the comparison of IV lidocaine versus placebo in managing patients undergoing thyroidectomy. The primary outcomes included postoperative pain by VAS measurements. Secondary outcomes included MAP, heart rate at extubation, and PONV. A random effects model was applied for the analysis, whereas statistical evaluation was done using RevMan 5.2.

**Results:**

A total of five RCTs of 375 patients were summarized, where 172 patients were in the lidocaine group and 203 in the placebo group. Significant reduction in postoperative pain of IV lidocaine was shown (SMD = −1.24; 95% CI: −2.7 to 0.30; *I*² = 97%, *p* = 0.11), heterogeneity was also reduced in a sensitivity analysis with *I*² = 46%. Extubation MAP and heart rate were also significantly lower (SMD for MAP = −1.27, *p* = 0.47; SMD for heart rate = −1.36, *p* = 0.11). PONV did not show any difference, whereas cough scores were remarkably improved (RR = 0.42, 95% CI = 0.28 to 0.63).

**Conclusion:**

IV lidocaine shows potential in reducing postoperative pain, improving hemodynamic stability, and decreasing cough incidence in thyroidectomy patients. However, its effect on PONV remains unclear, warranting further investigation.

**Abbreviations**
ATD: antithyroid drug
BMI: body mass index
CI: confidence interval
IL: interleukin
IV: intravenous
MAP: mean arterial pressure
NSAID: non‐steroidal anti‐inflammatory drug
PICOS: population, intervention, comparison, outcomes, and study design
PONV: postoperative nausea and vomiting
PRISMA: Preferred Reporting Items for Systematic Reviews and Meta‐Analyses
RCT: randomized controlled trial
RR: relative risk
SD: standard deviation
SMD: standard mean difference
TNF: tumor necrosis factor
VAS: Visual Analog Scale

## 1 Introduction

Thyroidectomy is a well‐documented and frequent operation in modern medicine that removes all or some parts of the thyroid gland that can be used to treat cancer, benign illness, or hormonal disorder that is resistant to medicinal treatment [1]. Thyroid surgery can be either complete, which removes the entire thyroid gland, or partial, which removes only a portion of the gland. Types of thyroidectomy include Total thyroidectomy, Partial thyroidectomy, Thyroid lobectomy, Isthmectomy, and Completion thyroidectomy [2]. Thyroidectomy may be performed in pathologies (both benign and malignant) like thyroid nodules, thyroid cancer, toxic adenomas, toxic multinodular goiter, obstructive or substernal goiter, primary thyroid lymphoma, hyperthyroidism, Grave's disease not curable through medical treatment [1]. Thyroidectomy is usually a safe operation but like any other operation, it also entails complications like infection, bleeding, hypoparathyroidism, and damage to vocal cords [3]. Historically, thyroidectomy was the preferred therapy for goiters but now improvements in diagnostic imaging and medical therapy have decreased the necessity for thyroidectomy for the majority of goiters and benign thyroid nodules [4]. When surgery is chosen as a therapy option, meticulous preoperative management is required to maximize surgical results. Pretreatment with Antithyroid drugs (ATDs) is indicated to quickly establish euthyroidism and reduce the possibility of a thyroid storm during surgery Methimazole, carbimazole, and propylthiouracil inhibit thyroid hormone release [5]. As for anesthesia, local anesthetics may be a choice like Mepivacaine and Lidocaine. Lidocaine is a local anesthetic substance extensively used for both local and topical anesthesia. Lidocaine prevents nerve depolarization by acting on sodium ion channels on the internal surface of nerve cell membranes. Lidocaine has a faster start of action with greater pKa values as compared to other local anesthetics [6]. Lidocaine can be given intramuscularly, intravenously, epidural, subarachnoid, and intrapleural. It is also utilized in central and peripheral nerve blocks, IV anesthesia applications, ventricular arrhythmias, and for post‐operative pain management by IV infusions [7]. Now it is demonstrated that intravenous IV lidocaine usage in the intraoperative stage decreases postoperative pain [8]. Patients who had lidocaine infusion had lower pain scores, postoperative analgesia and intraoperative anesthetic needs, quicker recovery of bowel function, and shorter hospital stays [9]. Lidocaine's analgesic, antihyperalgesic, and anti‐inflammatory characteristics make it useful in a variety of procedures [10]. Thyroid surgery often results in a high prevalence of postoperative nausea and vomiting [11]. It has been found that those patients who were administered with lidocaine had significantly lower risks for nausea and vomiting [12]. Intravenous Lidocaine not only reduced nausea and postoperative pain but also increased the recovery quality after thyroidectomy [13]. Hence, it is an excellent alternative approach that may be employed in thyroidectomy procedures as part of a multimodal analgesic strategy [14].

## 2 Methods

### 2.1 Eligibility Criteria

This meta‐analysis solely looks at randomized control trials (RCTs) to assess lidocaine's effectiveness as an analgesic for patients having thyroidectomies. (1) The first requirement for inclusion is that the participants have to have thyroidectomy. (2) The studies we incorporate ought to assess analgesic parameters or patient side effects. (3) Patients must be using lidocaine to reduce their pain; (1) Due to bias concerns, case reports, observational studies, and all other non‐randomized research are disqualified. (2) All in‐vitro research as well as studies conducted on species other than humans are not included. (3) Randomized trials that didn't fit our PICOS framework were disregarded, as can be seen in Supporting Information S1: Table 1.

### 2.2 Search Strategy and Data Sources

A search was done for randomized control trials published from July 2016 to May 2023. The only studies considered were those that were published in English. A methodical approach to database searching was devised to guarantee a comprehensive examination of pertinent literature. The search covered several databases, including Embase, Google Scholar, and PubMed. The foundation of the search method was the combination of free‐text terms and keywords from Medical Subject Headings with Boolean operators (AND, OR). Combining these terms made it easier to identify research on the effects of analgesia, thyroidectomy, and lidocaine.

### 2.3 Study Selection

This review has been published on Prospero. ID NO. CRD42024583226. To find research that might be relevant, we first looked through the titles and abstracts of the collected data. Then, a rigorous analysis of the full texts of the chosen publications was undertaken. This analysis was conducted independently by two researchers, aiming to determine their eligibility for inclusion in the meta‐analysis. In instances where disparities emerged, deliberations with a third researcher were undertaken to achieve consensus. In order to improve transparency in the review process, the PRISMA guidelines [15] were used to highlight the research selection process and the justifications for eliminating studies.

### 2.4 Risks of Bias Assessment

**Figure 1:**
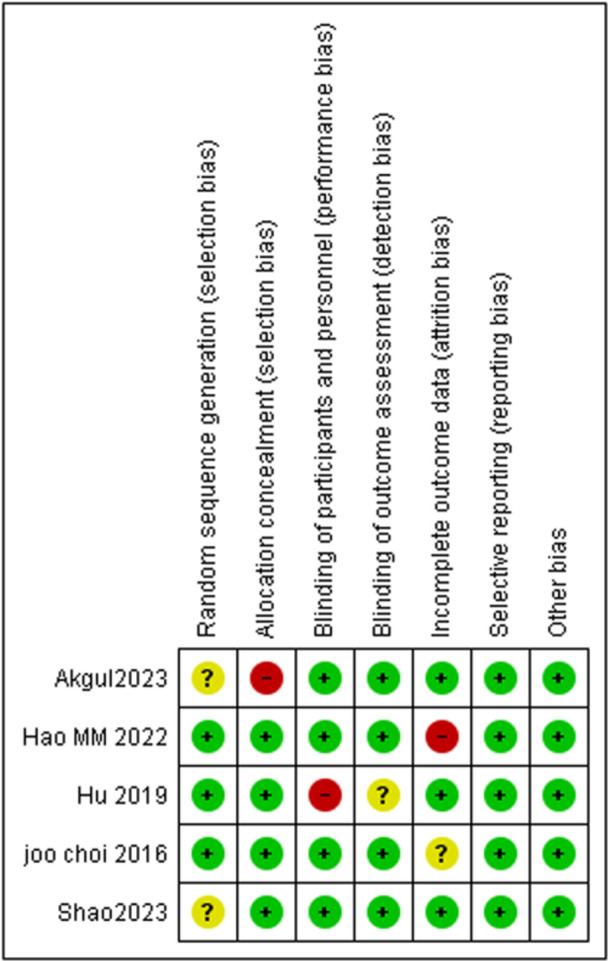
Summary of risk of bias.

We use the Cochrane Collaboration to perform a bias‐sensitive search of the randomized trials. The selected papers listed below were evaluated based on quality standards using this methodology. A variety of factors were considered while evaluating bias, including access to the outcome, blinding of outcomes, and selective reporting of results. The three categories into which the studies were separated are: indicates that certain outcomes are categorized as having an unclear risk of bias, a high risk of bias, or a low risk of bias due to insufficient data. To assess the potential for publication bias, a funnel graph was employed. Because of the rigorous technique used, it is possible to accurately assess the study's quality and identify any biases, leading to a trustworthy conclusion. Summary of risk of bias is given in Figure 1, and Supporting Information S1: Table 2.

### 2.5 Data Extraction

From the selected randomized control trial, we collect baseline data, information about the intervention, patient numbers, and continuous data. Data on the assessed pain metrics and the impact of lidocaine on individuals undergoing thyroid extirpation were gathered. This included details of any observed changes in pain, such as the VAS score for the postoperative pain measure. The postoperative nausea and vomiting, cough score, mean arterial pressure during extubation, heart rate during extubation, and awakening time were among the data collected about the side effects of lidocaine. Each study's general findings or inferences about the relationship between lidocaine and post‐thyroidectomy outcomes have been documented.

### 2.6 Statistical Analysis

We used RevMan (Review Manager version 5) to evaluate the data in this meta‐analysis. For every statistical analysis, a random effects model was used. The main aim of the study was to evaluate the effectiveness of lidocaine use in thyroidectomy patients related to analgesia and the side effect profile of participants in the chosen clinical trials using quantitative methods. For the analgesia and adverse effects outcomes, we determined the mean and standard deviation (SD) values from each trial that was part of the review. These means and standard deviations were then input into RevMan 5. We computed the risk difference (RD) or relative risk (RR) using a 95% confidence interval (CI) for dichotomous data. We utilized a standard mean difference (SMD) and a 95% confidence interval (CI) for continuous data. A p‐value of less than 0.05 was considered statistically significant for the results. To visualize the data and evaluate statistical heterogeneity, we used a forest plot and the *χ* ^2^ and *I* ^2^ tests. If the *p* value was less than 0.05 or the *I* ^2^ exceeded 50%, significant heterogeneity was deemed to exist. When there was a lot of variation, we used the leave‐one‐out method to figure out where it came from. This methodical approach guarantees the precision of our findings and fosters trust in their interpretation.

## 3 Results

### 3.1 Study Selection and Characteristics

**Figure 2:**
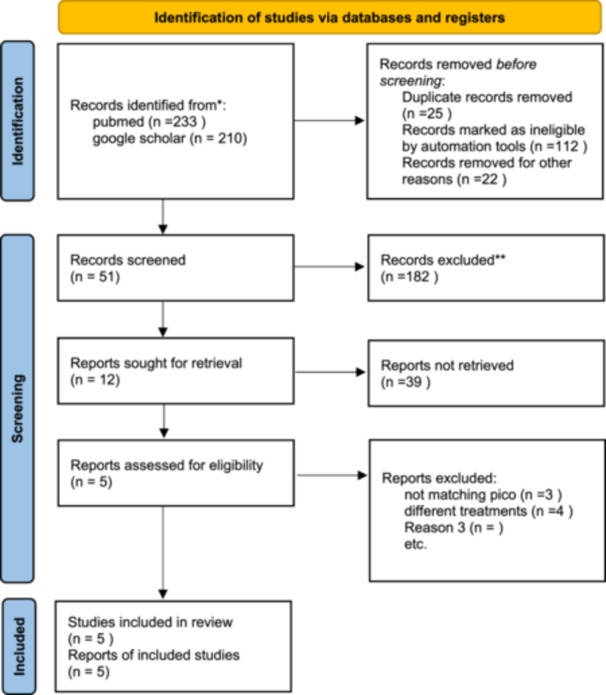
PRISMA flow chart. *Consider, if feasible to do so, reporting the number of records identified from each database or register searched (rather than the total number across all databases/registers). **If automation tools were used, indicate how many records were excluded by a human and how many were excluded by automation tools. This work is licensed under CC BY 4.0. To view a copy of this license, visit https://creativecommons.org/licenses/by/4.0/. *Source:* Page MJ, et al. BMJ 2021;372:n71. doi: 10.1136/bmj.n71.

After the initial exhaustive literature search yielding studies including 5 RCTs [14, 16, 17, 18, 19] were selected and included in the meta‐analysis. A more detailed overview of the process is represented in the PRISMA flowchart in Figure 2 Outcome data was extracted and pooled for a total of 375 Patients of which 172 were stratified to receive lidocaine and 173 to receive placebo. The mean follow‐up duration between the included studies was weeks with a mean age of 45.91 ± 6.4 and a mean BMI of 23.8 ± 1.72. A detailed overview of the study and baseline characteristics is given in detail in Table 1.

**Table 1:** Baseline and study detail.

Baselines and study detail									
Year	Doi	Study design	Author	Country	Age	*n*(F/M)	No. of PT in INTRV	No. of PT in CNTRL	BMI
2023	· 10.1590/1806‐9282.20220681	rct	Emrah Akgul	Turkey	48.15 ± 7.48	40 (32/8)	20	20	27.74 ± 2.32
2023	· 10.1186/s12871‐023‐02109‐w	rct	Caiqun Shao	China	37.3 ± 4.95	68 (68/0)	33	35	21.03 ± 1.45
2016	10.1007/s00268‐016‐3619‐6	rct	Geun Joo Choi	Korea	50.25 ± 8.62	56 (43/13)	28	28	22.83 ± 1.40
2022	10.1097/AJP.0000000000001027	rct	Guo, Hao MM	China	45 ± 5.35	61 (41/20)	31	30	NA
2019	10.1186/s12871‐019‐0739‐1	rct	Shenghong Hu	China	48.85 ± 5.68	120 (69/51)	60	60	NA

### 3.2 Risk of Bias Assessment

All studies included in this meta‐analysis had a low overall risk of bias. A detailed overview of individual risk of bias assessment is presented in Supporting Information S1: Figure 1.

### 3.3 Primary Outcome

#### 3.3.1 VAS Score

**Figure 3:**
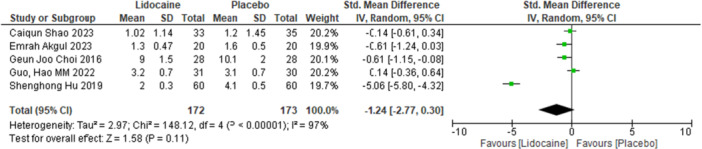
Forest plot of VAS score before leaving one out analysis.

Our primary outcome “VAS score” was reported by 5 studies. Pooling data was analysed indicated that there is no significant difference between the two groups in changing VAS score in patients stratified to lidocaine during thyroidectomy when compared with placebo. [(SMD) = −1.24, 95% confidence interval (CI) = (−2.7 to 0.30), *I* ^2^ = 97%, *p*= 0.11]. There was significant heterogeneity among the studies, as evidenced by an I2 value of 97%. The forest plot for the VAS score is presented in Figure 3.

#### 3.3.2 Leave One Out Analysis

**Figure 4:**
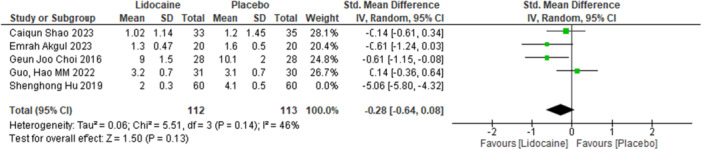
Forest plot of VAS score after sensitivity analysis.

Due to significant heterogeneity, we performed a sensitivity analysis to evaluate its cause. After excluding the Sheng Hong Hu 2019 study, our heterogeneity dropped from 97% to 46%. The forest plot for the VAS score after leaving one out analysis is presented in Figure 4.

### 3.4 Secondary Outcomes

#### 3.4.1 Map During Extubation

**Figure 5:**
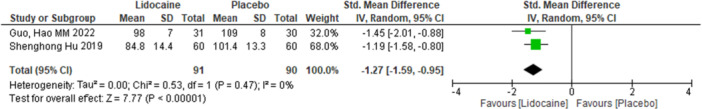
Forest plot of MAP during extubation.

A decrease in mean arterial pressure was noted in participants given lidocaine compared to those given a placebo when an analysis using data from two studies was performed. No significant heterogeneity was noted [SMD = −1.27, 95% CI = (−1.59 to −0.95), *I* ^2^ = 0%, *p *< 0.00001]. A forest plot for the MAP during extubation is presented in Figure 5.

#### 3.4.2 Heart Rate During Extubation

Heart rate during extubation was also an outcome of interest in our selected studies, and upon analyzing changes, a statistically significant decrease was evident in the lidocaine group as compared to the placebo. [SMD = −1.36, 95% CI = (−1.92 TO −0.80), *I* ^2^ = 62%, *p *< 0.00001]. The forest plot for heart rate during extubation is presented in Supporting Information S1: Figure 2.

#### 3.4.3 Awakening Time

Two studies were evaluated for the awakening time in the intervention and control groups, and there was no difference in the outcomes of the intervention group and the control group. The reported results came to be non‐significant. Besides, a high heterogeneity was noted in the analytical data [SMD = −0.22, 95% CI = (−1.92 to 1.13), *I* ^2^ = 94%, *p *= 0.75]. The forest plot for awakening time is presented in Supporting Information S1: Figure 3.

#### 3.4.4 PONV

The analyzed data showed no significant difference in postoperative nausea and vomiting risk between the intervention and control groups. However, the overall result was non‐significant. [RR = 0.58, 95% CI = (0.33 to 1.01), *I* ^2^ = 0%, *p* = 0.06]. The forest plot for PONV is presented in Supporting Information S1: Figure 4.

#### 3.4.5 Cough Score

The cough score noted in the patients receiving lidocaine was non‐significant compared to the control group. [RR = 0.42,95% CI = (0.28 to 0.63), *I* ^2^ = 0%, *p* = 0.06]. The forest plot for cough score is presented in Supporting Information S1: Figure 5.

## 4 Discussion

Our original meta‐analysis aimed to evaluate the efficacy of lidocaine in managing pain and other hemodynamic responses in patients undergoing thyroidectomy. The meta‐analysis incorporated five randomized controlled trials (RCTs), pooling data from 375 patients—172 of whom received lidocaine, while 173 received a placebo. The primary outcome of interest was the Visual Analog Scale (VAS) score, a widely accepted metric for assessing pain intensity. In contrast, secondary outcomes included mean arterial pressure (MAP) during extubation, heart rate during extubation, awakening time, postoperative nausea and vomiting (PONV), and cough score. Regarding the primary outcome, the meta‐analysis demonstrated a non‐significant reduction in VAS scores for patients administered lidocaine compared to those who received a placebo. For the secondary outcomes, the results were more mixed. A significant decrease in MAP during extubation was observed in patients treated with lidocaine compared to the placebo. Similarly, the heart rate during extubation was significantly lower in the lidocaine group. On the other hand, the awakening time did not differ significantly between the intervention and control groups. Furthermore, the analysis of PONV and cough scores revealed that while lidocaine significantly reduced the cough scores, it did not significantly impact PONV rates. Most of the outcomes were significant, particularly for hemodynamic parameters and cough scores, favoring lidocaine over placebo. However, the non‐significance in VAS score and PONV suggests that lidocaine efficacy may be more pronounced in controlling hemodynamic responses rather than in managing pain and nausea/vomiting post thyroidectomy.

Lidocaine, a local anesthetic from the amide class, is well known for its effects on both the nervous and immune systems [20]. It offers several therapeutic benefits, including analgesia, anti‐hyperalgesia and anti‐inflammatory properties. Clinically, lidocaine can be administered through various routes such as epidural, subarachnoid, intrapleural, intravenous, intramuscular, intraarticular, and topical applications [7]. Like other local anesthetics, lidocaine provides local or regional anesthesia by reversibly blocking nerve impulse conduction [7].

Although the pain following thyroidectomy is not as intense as that experienced after major surgeries, it is still important to address postoperative pain. Pain perception is subjective and varies significantly among individuals making pain management crucial [21].

The primary sources of post‐thyroidectomy pain include neck incision, cervical hyperextension, trauma from orotracheal intubation, and the insertion of surgical drains [22]. Several studies have demonstrated that the use of intravenous lidocaine during the intraoperative period can reduce postoperative pain [8, 9]. Our findings, which show a reduction in VAS scores following lidocaine administration during thyroidectomy, are consistent with previous studies where IV lidocaine was effective in pain reduction. The mechanisms underlying these effects include the attenuation of neuronal responses to postoperative pain by blocking nerve conduction suppressing central sensitization and reducing the release of peripheral inflammatory mediators [23, 24].

Thyroid surgery is also associated with a high incidence of post‐operative nausea and vomiting (PONV) [25]. The exact mechanism behind PONV following thyroidectomy remains unclear but they may involve inflammatory responses due to surgical trauma to neck structure and vagal nerve stimulation [11]. PONV complications include wound bleeding, hematoma formation, and airway obstruction [25]. Lidocaine when administered shows anti‐PONV effects and its actions may be attributed to improved gastrointestinal recovery and an opioid‐sparing effect [26].

Given the thyroid gland's rich vascular supply and high blood perfusion, postoperative bleeding is more common after thyroid surgery than after the surgical procedure. such bleeding typically occurs within 12 h postoperatively, especially within the first 6 h [27]. Coughing may increase the risk of postoperative bleeding. While suction drains are commonly used in thyroidectomy, they are more effective in removing blood than preventing bleeding. post‐thyroidectomy bleeding remains a significant concern, often leading to severe complications such as cervical hematoma, reoperation, and even cardiac arrest [28]. Intravenous lidocaine has effectively reduced cough and stabilized hemodynamic changes during tracheal extubation in patients undergoing thyroid surgery. Participants administered lidocaine during extubation exhibited a significant decrease in mean arterial pressure and heart rate, likely due to lidocaine's ability to modulate autonomic responses by inhibiting nerve activity and reducing sympathetic nervous system output. This stabilization of hemodynamic parameters helps minimize stress on the surgical, thereby reducing the complication. The precise mechanism by which lidocaine suppresses coughing is not entirely understood. The vagus nerve, which extensively innervates the larynx, trachea, extrapulmonary and intrapulmonary bronchi, and lung parenchyma, is stimulated by mechanical events related to secretion and edema from airway inflammation, leading to coughing. As a non‐selective inhibitor of voltage‐gated sodium channels, Lidocaine prevents initiating and conducting action potentials to the central nervous system [29].

Surgical trauma triggers an inflammatory response, leading to the release of cytokines like IL‐1, IL‐6, and TNF which can cause sleep disturbance after surgery. Lidocaine helps control this by lowering the release of such cytokines, reducing inflammation, and improving postoperative sleep [20, 30].

Opioids when given perioperatively influence immune function and have been shown to suppress multiple aspects of immune functions, including both humoral and cellular immunity. Apart from lidocaine, many other non‐opioids are options available as an alternative for thyroidectomy [31].

NSAIDs are effective non‐opioid alternatives that significantly reduce pain scores. however, caution is needed in patients with risk factors such as renal issues, allergies, and gastrointestinal concerns like bleeding and ulceration [31]. Gabapentin has shown significant benefits in reducing postoperative pain and the need for additional analgesics, especially within the first 24 h after surgery. they are effective in managing both acute and chronic postoperative pain [31]. Ketamine is a potential non‐opioid analgesic option, although its use in thyroid surgery requires further research due to limited studies [31]. Acetaminophen is recognized as an effective postoperative analgesic, endorsed by the American Pain Society. However, its effectiveness in thyroid surgery is debated, with some evidence showing reduced pain and less need for rescue analgesics. At the same time, other data suggest it may be less effective compared to NSAIDs [31].

The VAS score exhibited a heterogeneity of almost 97%. However, the substantial reduction from 97% to 46% after excluding the study by Shenghong Hu suggests that this study contributed significantly to the overall inconsistency among the studies we included in our meta‐analysis. A critical examination of ethics reveals that Shenghong Hu's study differed in several key aspects. Notably, it employed a higher dose of lidocaine and dexmedetomidine along with a distinct anesthesia management protocol involving propofol and remifentanil which likely influenced postoperative pain outcomes differently compared to other studies. Additionally, the study's larger sample size and potential variability in surgical techniques, as three different surgeons performed all surgeries, could have contributed to the higher heterogeneity.

Although some outcomes did not reach statistical significance, this may reflect the limited statistical power due to the small sample size of the included studies. However, the observed reductions in postoperative mean arterial pressure and heart rate may still hold clinical significance, as even modest improvements in these parameters can enhance patient recovery and safety in thyroidectomy. Hence, Therefore, future studies with larger sample sizes are warranted to confirm these trends and better delineate both the statistical and clinical significance of intraoperative lidocaine use.

Our meta‐analysis has several limitations that need to be acknowledged. Firstly, the small sample size across the included studies might affect the reliability of our results. Additionally, the absence of subgroup data prevented us from conducting subgroup analysis, which could have provided more nuanced insights. Furthermore, we didn't perform meta‐regression due to the limited sample size, which restricted our ability to assess the influence of specific study characteristics on the overall effect size.

## 5 Conclusion

In conclusion, Intravenous lidocaine effectively decreases postoperative pain, stabilizes hemodynamic parameters, and reduces cough scores in thyroidectomy patients. However, its impact on PONV remains inconclusive, necessitating further research.

## Author Contributions

**Ayesha Khan:** conceptualization, resources, project administration, writing – review and editing, writing – original draft, investigation, methodology, formal analysis, data curation, supervision, validation, visualization. **Syeda Emmama, Mohnoor Aamir, Munazzah Marium, Iftikhar Rashid:** data curation, writing – original draft, methodology. **Marium Abbas, Aleezah Fatima, Alina Amir, Shafin Bin Amin:** visualization and methodology. **Syed Ali Arsal:** data curation, writing – review and editing, writing – original draft, methodology. **Umer Iqbal, Muhammad Maaz, Ashish Kumar:** visualization and methodology.**Inibehe Ime Okon:** supervision, writing – review and editing, funding acquisition, validation.

## Funding

The authors received no specific funding for this work.

## Ethics Statement

The authors have nothing to report.

## Consent

The authors have nothing to report.

## Conflicts of Interest

The authors declare no conflicts of interest.

## Transparency Statement

The lead author Inibehe Ime Okon affirms that this manuscript is an honest, accurate, and transparent account of the study being reported; that no important aspects of the study have been omitted; and that any discrepancies from the study as planned (and, if relevant, registered) have been explained.

## Supporting information


**Supplementary Table: 01:** Search strategy table. **Supplementary Table: 02:** Risk of Bias Assessment by Cochrane Risk of Bias Tool. **Supplementary figure: no 1:** Individual risk of bias assessment. **Supplementary figure: no. 2:** Forest plot of heart rate during extubation. **Supplementary figure: no. 3:** Forest plot of awakening time. **Supplementary figure: no. 4:** Forest plot of PONV. **Supplementary figure: no. 5:** Forest plot of cough score.

## Data Availability

The authors have nothing to report.
