# Supplementary material for: A Potential Role of Intraoperative Lidocaine in Patients Undergoing Thyroidectomy—An Original Systematic Review and Meta‐Analysis
Source: Health Sci Rep. 2025 Dec 22;8(12):e71675. doi: 10.1002/hsr2.71675 (PMC12723206; doi:10.1002/hsr2.71675)
Supplement: Supplementary file 1 — Supplementary Table: 01: Search strategy table. Supplementary Table: 02: Risk of Bias Assessment by Cochrane Risk of Bias Tool. Supplementary figure: no 1: Individual risk of bias assessment. Supplementary figure: no. 2: Forest plot of heart rate during extubation. Supplementary figure: no. 3: Forest plot of awakening time. Supplementary figure: no. 4: Forest plot of PONV. Supplementary figure: no. 5: Forest plot of cough score. [file HSR2-8-e71675-s001.docx]

**For review and publication:**

**Supplementary figure 1.** Individual risk of bias assessment**.**


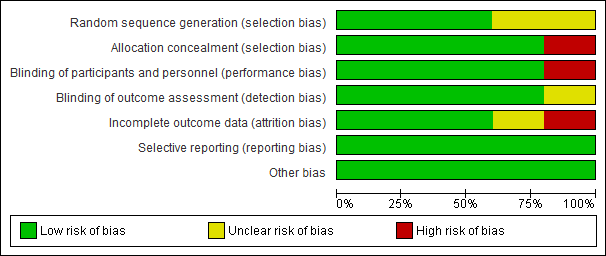


**Supplementary figure 2.** Forest plot of heart rate during extubation**.**

**
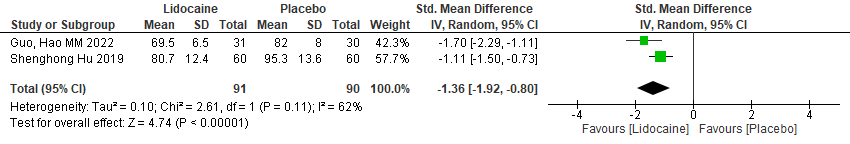
**

**Supplementary figure 3.** Forest plot of awakening time**.**

**
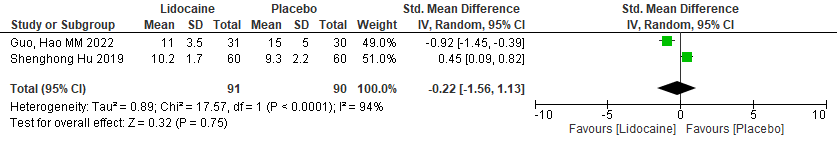
**

**Supplementary figure 4.** Forest plot of PONV**.**


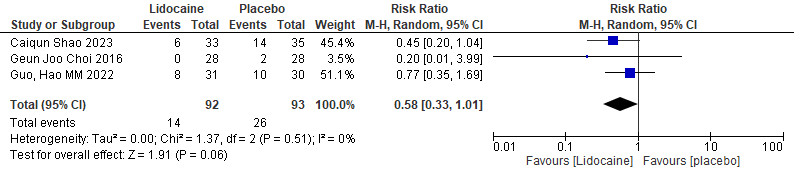


**Supplementary figure 5** Forest plot of cough score**.**


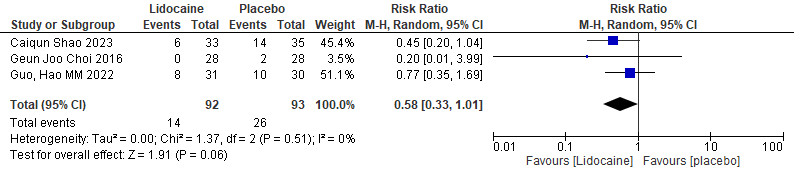


**Supplementary table 1.** Search strategy.

| **Search engine** | **Search strategy** | **Results** |
| --- | --- | --- |
| Pubmed | ((((("analgesic s"[All Fields] OR "analgesically"[All Fields] OR "analgesics"[Pharmacological Action] OR "analgesics"[MeSH Terms] OR "analgesics"[All Fields] OR "analgesic"[All Fields]) AND ("efficacies"[All Fields] OR "efficacious"[All Fields] OR "efficaciously"[All Fields] OR "efficaciousness"[All Fields] OR "efficacy"[All Fields])) OR ("pain management"[MeSH Terms] OR ("pain"[All Fields] AND "management"[All Fields]) OR "pain management"[All Fields])) AND ("lidocain"[All Fields] OR "lidocaine"[MeSH Terms] OR "lidocaine"[All Fields] OR "lignocaine"[All Fields] OR "lidocaine s"[All Fields] OR "lignocain"[All Fields])) OR ("lidocain"[All Fields] OR "lidocaine"[MeSH Terms] OR "lidocaine"[All Fields] OR "lignocaine"[All Fields] OR "lidocaine s"[All Fields] OR "lignocain"[All Fields]) OR ("anesthetics local"[Pharmacological Action] OR "anesthetics, local"[MeSH Terms] OR ("anesthetics"[All Fields] AND "local"[All Fields]) OR "local anesthetics"[All Fields] OR ("local"[All Fields] AND "anesthetic"[All Fields]) OR "local anesthetic"[All Fields] OR "anesthesia, local"[MeSH Terms] OR ("anesthesia"[All Fields] AND "local"[All Fields]) OR "local anesthesia"[All Fields])) AND ("thyroidectomy"[MeSH Terms] OR "thyroidectomy"[All Fields] OR "thyroidectomies"[All Fields]) | 233 |
| Google scholar | (((((analgesic efficacy) OR (pain management)) AND (lidocaine)) OR (lignocaine)) OR (local anesthetic)) AND (thyroidectomy) | 210 |
| Embase | (((((analgesic efficacy) OR (pain management)) AND (lidocaine)) OR (lignocaine)) OR (local anesthetic)) AND (thyroidectomy) | 200 |

**Supplementary table 2.** Risk of Bias Table.

**Analgesic Efficacy of Intraoperative Lidocaine In Patient Undergoing Thyroidectomy**

| **STUDIES** | **COCHRANE RISK OF**  **BIAS TOOL BIAS** | **RISK OF BIAS** | **AUTHOR JUDGMENT** |
| --- | --- | --- | --- |
| **Akgul**  **2023** | **random sequence generation (selection bias)**  **allocation concealment (selection bias)**  **blinding of participants and personnel (performance bias)**  **blinding of outcome assessment (detection bias)**  **incomplete outcome data ( attrition bias)**  **selective reporting ( reporting bias)**  **other bias** | **unclear risk**  **high risk**  **low risk**  **low risk**  **low risk**  **low risk**  **low risk** | **Quote: "The study address randomization unclearly."**  **Quote: "There was lack of accuracy in allocation."**  **Quote: " Patients were properly blinded along with doctors."**  **Quote: "The operators who assessed the outcomes were unaware of which treatment each patient had received."**  **Quote: "All the data regarding outcome was available."**  **Quote: " All outcomes were properly reported."**  **Quote: " This study had no other bias."** |
| **Hao MM**  **2022** | **random sequence generation (selection bias)**  **allocation concealment (selection bias)**  **blinding of participants and personnel (performance bias)**  **blinding of outcome assessment (detection bias)**  **incomplete outcome data ( attrition bias)**  **selective reporting ( reporting bias)**  **other bias** | **low risk**  **low risk**  **low risk**  **low risk**  **high risk**  **low risk**  **low risk** | **Quote: " Patients were randomly assigned to two groups."**  **Quote: "Patients were centrally allocated."**  **Quote: "Doctors were not acknowledged regarding the groups."**  **Quote: " The outcome assessors were also blinded to treatment allocation."**  **Quote:** " Insufficient detail provided for judgement."  **Quote: " All pre- specified end points were reported."**  **Quote: " The study appears to be free of other sources of bias."** |
| **Hu**  **2019** | **random sequence generation (selection bias)**  **allocation concealment (selection bias)**  **blinding of participants and personnel (performance bias)**  **blinding of outcome assessment (detection bias)**  **incomplete outcome data ( attrition bias)**  **selective reporting ( reporting bias)**  **other bias** | **low risk**  **high risk**  **unclear risk**  **low risk**  **low risk**  **low risk**  **low risk** | **Qoute: "The study follows randomization properly."**  **Quote: " The allocation was not centrally web based."**  **Quote: "The study contains exposure measurement with explicit indication of performance."**  **Qoute: "The outcomes were assessed and analysed by independent technicians who were blind to clinical and interventional information."**  **Quote: " Insufficient detail provided for judgement."**  **Quote: "All outcome data were present."**  **Quote: "The study appears to be free of other bias."** |
| **joo choi**  **2016** | **random sequence generation (selection bias)**  **allocation concealment (selection bias)**  **blinding of participants and personnel (performance bias)**  **blinding of outcome assessment (detection bias)**  **incomplete outcome data ( attrition bias)**  **selective reporting ( reporting bias)**  **other bias** | **low risk**  **low risk**  **low risk**  **low risk**  **unclear risk**  **low risk**  **low risk** | **Qoute: "The study follows randomization properly."**  **Quote: " Centrally allocated web based."**  **Quote: "The study focused on exposure measurement without explicit indication of performance."**  **Qoute: "The outcomes were assessed and analysed by independent technicians who were blind to clinical and interventional information."**  **Quote: " Insufficient detail provided for judgement."**  **Quote: "All pre- specified end points were reported."**  **Quote: "The study appears to be free of other bias."** |
| **Shao**  **2023** | **random sequence generation (selection bias)**  **allocation concealment (selection bias)**  **blinding of participants and personnel (performance bias)**  **blinding of outcome assessment (detection bias)**  **incomplete outcome data ( attrition bias)**  **selective reporting ( reporting bias)**  **other bias** | **unclear risk**  **low risk**  **low risk**  **low risk**  **low risk**  **low risk**  **low risk** | **Quote: "There was not proper enrollment of consecutive patients."**  **Quote: " There was lack of accuracy in allocation."**  **Quote: " Patients were properly blinded along with doctors."**  **Quote: " The study mentions that it was conducted in a double- blind fashion."**  **Quote: " All outcome data was present."**  **Quote: "All outcomes were properly reported."**  **Quote: "The study appears to be free of other bias."** |
